# Leptospirosis: First Reported Case in Lebanon

**DOI:** 10.7759/cureus.95868

**Published:** 2025-10-31

**Authors:** Georges Khalil, Youssef El Toum, Elissa El Toum

**Affiliations:** 1 Medical Microbiology, Saint Joseph University, Beirut, LBN; 2 Faculty of Medicine, Saint Joseph University, Beirut, LBN; 3 Neurology, Saint George Hospital University Medical Center, Beirut, LBN

**Keywords:** fever, jaundice, leptospirosis, rodent, zoonotic infection

## Abstract

Leptospirosis is a zoonosis transmitted in warm and humid climates. It is contacted by humans through direct contact with the urine of infected hosts, mainly rodents, or via contaminated water. The clinical presentation ranges from asymptomatic or mild disease to severe multi-organ failure that may be fatal.

We present the case of a 42-year-old man who presented with two weeks of fever, headache, abdominal pain, and myalgia. On admission, he was hypotensive, febrile, and jaundiced. Leptospirosis was suspected in this patient and later confirmed after early initiation of treatment.

This case highlights the occurrence of leptospirosis in Lebanon, a condition that may go underrecognized and subsequently patients may receive inadequate care. Early recognition and prompt treatment are crucial to ensure better outcomes, especially in severe cases.

## Introduction

Leptospirosis is a zoonosis transmitted by the spirochete Leptospira and constitutes a risk to human and animal health [1]. It is mainly transmitted to humans by rodents, in particular rats by direct contact with infected urine or through indirect contact with contaminated water, such as rivers, even in the absence of skin abrasions in cases of prolonged submersion [1]. Leptospirosis is not transmitted from human to human [1].

The incidence of Leptospirosis is increasing globally on a large scale, and it is more frequent in tropical regions and in warm and humid climates [2]. When adjusted for age and gender demographics, the estimated annual morbidity and mortality of leptospirosis in Lebanon are 2.93 cases (95% CI: 0.92-5.37) and 0.15 deaths (95% CI: 0.05-0.26) per 100,000 individuals, respectively [3]. However, the epidemiological profile of leptospirosis in Lebanon remains poorly defined, as no case reports have been published to date [3].

The clinical presentation of leptospirosis is variable and depends on the host. In most cases, it is asymptomatic or self-limited, while some cases might be fatal [4]. Symptomatology is mainly classified into the icteric or anicteric form [5]. Patients with the non-icteric form, the most common form, typically present with non-specific symptoms such as fever, rigors, myalgias and headache. This form is often self-limited but may progress into a more severe icteric form with a mortality rate of 5-15% [5]. Consequently, treatment with antibiotics should be initiated as soon as the infection is suspected.

In Lebanon, the warm and humid climate and the presence of potential maintenance hosts such as rodents create a favorable environment for the transmission of leptospirosis [6-7]. Nevertheless, the disease remains neglected in the nation [3]. We report the first described case of leptospirosis in Lebanon.

## Case presentation

A 42-year-old male with a past medical history of intravenous (IV) drug use and epilepsy was admitted to the Department of Infectious Diseases with a two-week history of fever, headache, abdominal pain, and myalgia. On admission, he was hypotensive with a blood pressure of 87/50 mmHg, febrile (39.3 °C) and presented icterus sclera. The patient never traveled abroad.

Initial laboratory investigations showed hemoglobin at 9.8 g/dL, leukopenia with a white blood cell count of 3,300/µL, elevated C-reactive protein at 156 mg/dL and thrombocytopenia with platelets at 35,000/µL. Liver function tests revealed serum glutamic oxaloacetic transaminase (SGOT) 40.5 IU/L, serum glutamic pyruvic transaminase (SGPT) 32.6 IU/L, Gamma GT 189 IU/L, alkaline phosphatase 304 IU/L, total bilirubin 29 mg/dL (direct bilirubin 21.4 mg/dL), and lipase 16.6 U/L (Table 1). Electrolytes showed hyponatremia (Na 127.9 mmol/L) and mild hypokalemia (K 3.35 mmol/L).

**Table 1:** Laboratory tests on admission and days later CRP: C-reactive protein; ASAT: Aspartate aminotransferase; ALAT: Alanine aminotransferase; Gamma GT: Gamma-Glutamyl transferase; INR: International Normalized Ratio. - indicates that the laboratory test has not been performed on the specified day.

	Day 1	Day 4	Day 7	Reference Range
Hemoglobin (g/dL)	9.8	8.3	9.6	13 – 17.7
White Blood Cells (/µL)	3300	7400	13000	4000 – 10000
Platelets (/µL)	35000	41000	197000	150000 – 500000
Creatinine (mg/dL)	0.855	0.443	0.436	0.7 – 1.2
CRP (mg/L)	156	87.2	21.3	<3
ASAT (IU/L)	40.5	-	15.7	<29
ALAT (IU/L)	32.6	-	22.5	<42
Alkaline Phosphatase (IU/L)	304	-	208	<128
Gamma GT (IU/L)	189	-	181	<43
Total bilirubin (mg/dL)	29	-	7.6	2 – 12
Direct bilirubin (mg/dL)	21.4	-	5.84	0 – 3
INR	100	-	100	100

**Figure 1:**
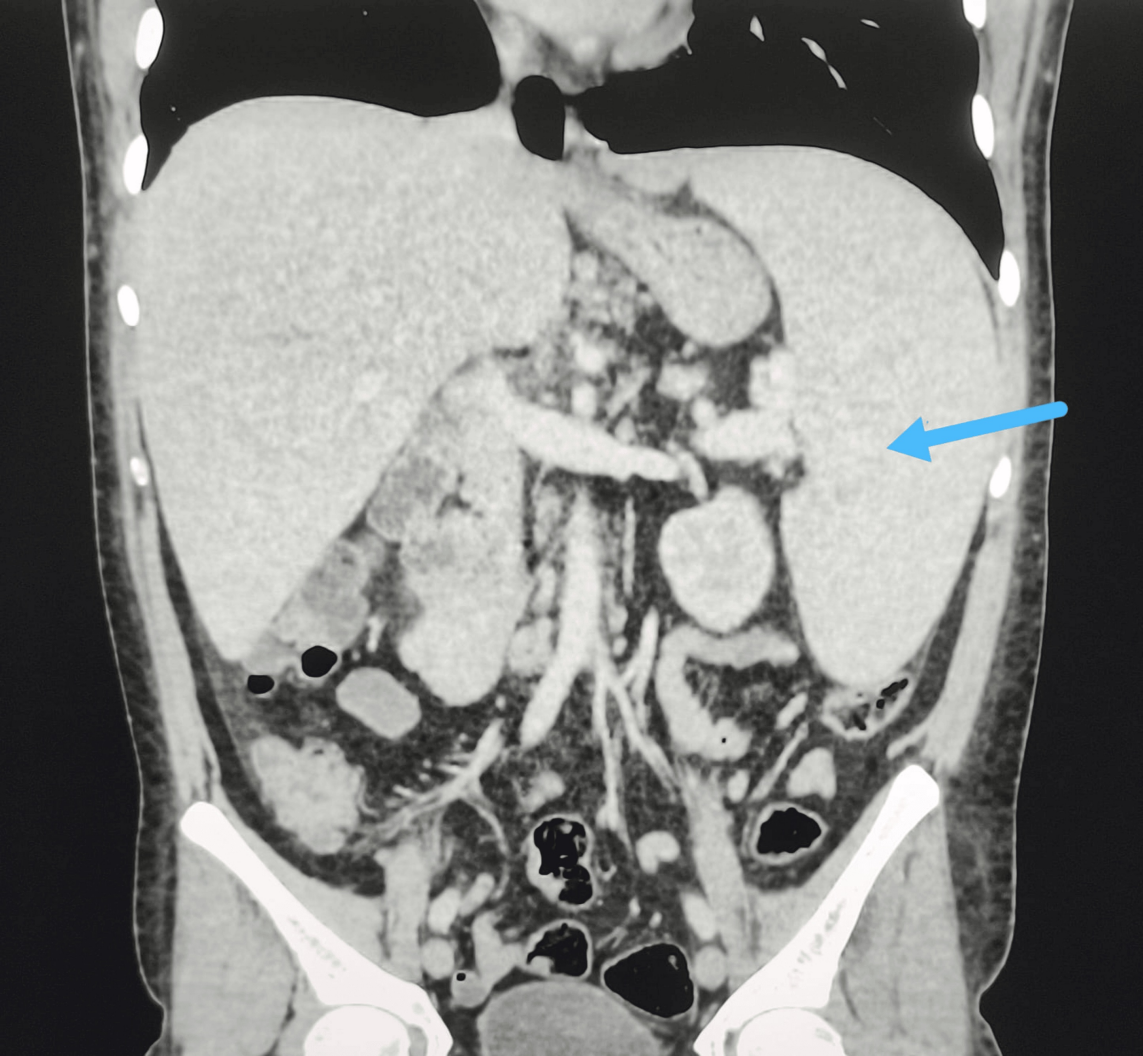
Abdominal CT scan demonstrating homogeneous splenomegaly The blue arrow indicates the enlarged spleen of the patient.

**Figure 2:**
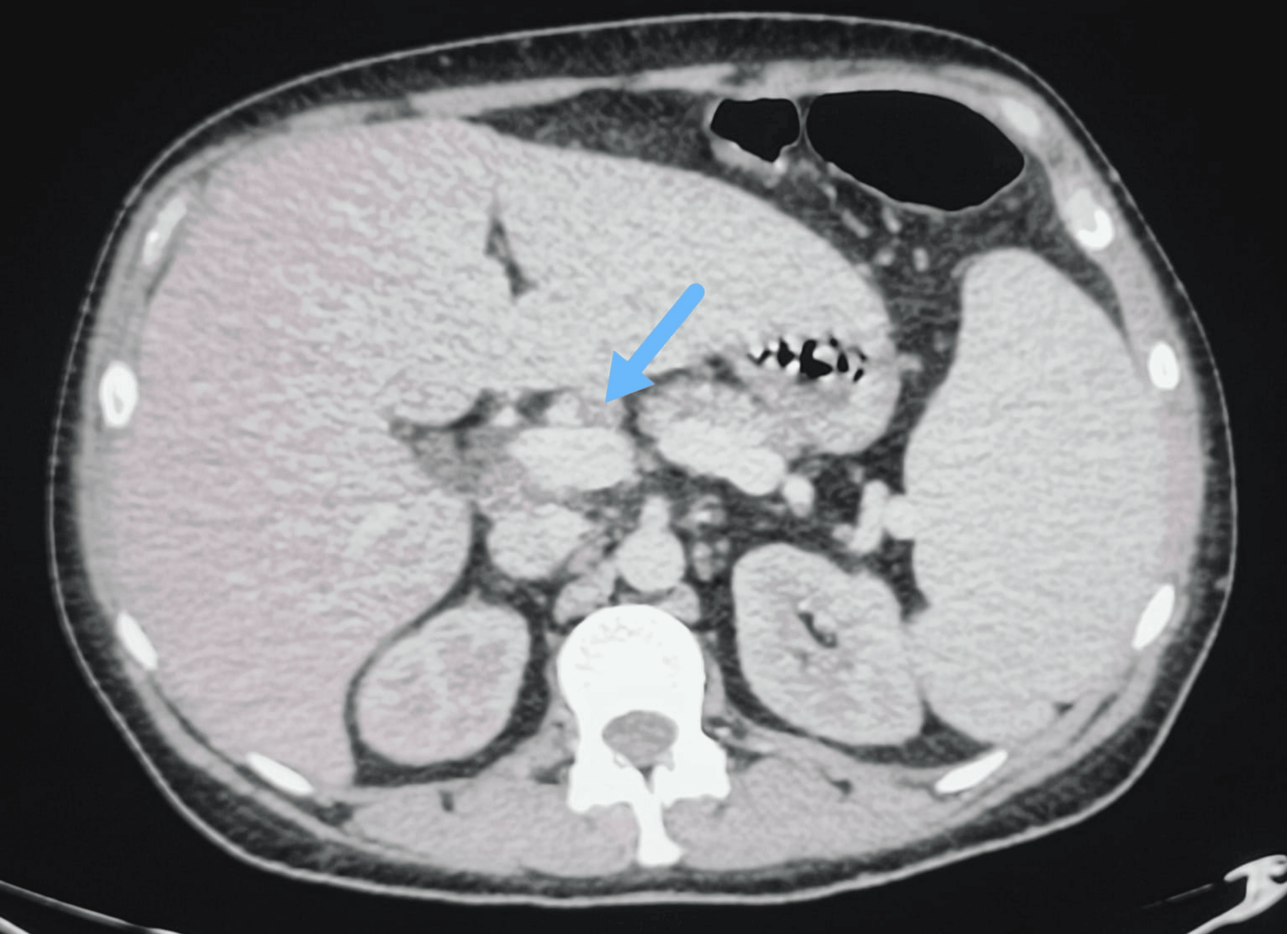
Abdominal CT scan demonstrating perihepatic lymph nodes The blue arrow indicates a perihepatic lymph node.

Abdominal CT scan demonstrated homogeneous splenomegaly (Figure 1), as well as multiple periportal and perihepatic lymph nodes, the largest measuring 18 mm in short axis within the portocaval space (Figure 2). The CT did not reveal any signs of chronic hepatic failure, biliary duct obstruction, choledocholithiasis, or cholecystitis.

Blood cultures were performed several times and remained sterile. PCR testing was conducted in search of multiple organisms (Plasmodium falciparum, Plasmodium malariae, Plasmodium vivax, Plasmodium ovale, Salmonella enterica, Dengue virus, Chikungunya virus, West Nile virus), all of which returned negative.

The patient reported that his dog had died two weeks earlier from leptospirosis, confirmed by urine PCR. In light of this history, leptospirosis was strongly suspected, and the patient was promptly started on intravenous ceftriaxone (2g once daily) for one week. A blood PCR for leptospirosis was performed two days after the start of antibiotics and returned negative. An IgM ELISA sample was also sent to a reference laboratory in France.

Within several days, the fever and associated symptoms were resolved, and the patient was subsequently discharged. Two weeks later, serological testing revealed a Leptospira IgM level of 61 IU/mL, with an IgM control level 10 days later of 240 IU/ML, confirming an acute leptospirosis infection.

The non-suspicion of the infection may have led to a bitter prognosis and possibly to the patient's death, especially since the patient had the icteric form of the disease.

## Discussion

This case represents the first confirmed report of leptospirosis in Lebanon. Despite the country’s warm and humid climate and the presence of rodent reservoirs, leptospirosis remains largely underrecognized, highlighting the need for heightened clinical awareness. Leptospirosis is a zoonosis transmitted to humans through direct or indirect contact with the urine of infected animals, particularly rodents, in warm and humid environments. Leptospira interrogans, which is the most likely source behind leptospirosis in humans, has been detected in sewer rats in Beirut [3,8].

Thus, although the climate of Lebanon is favorable for its transmission and a favorable host is present for the transmission, the disease’s epidemiology remains poorly defined [3]. This case represented the first confirmed instance of the disease, implying the presence of the infection in Lebanon. Therefore, leptospirosis should be considered in patients presenting with a fever of unknown origin, particularly when associated with renal failure and jaundice, as a misdiagnosis may be fatal [5].

The presentation of leptospirosis is classified into non-icteric and icteric forms. Patients with the non-icteric form present with abrupt onset of fever, rigors, myalgias (especially in the calves and lower back) and headache in 75-100% of cases [5]. About 50% of patients experience nausea, vomiting and diarrhea, and a nonproductive cough occurs in 25 to 35% of patients [9]. Arthralgias, bone pain, abdominal pain and sore throat are less common symptoms [9].

Alternatively, icteric leptospirosis occurs in 5-10% of patients and has a mortality rate of 5-15%. This form is typically characterized by fever, jaundice and multiple organ involvement [5]. It can lead to renal failure with hematuria and oliguria, or liver necrosis with tender hepatomegaly, normal or slightly elevated cytolytic liver enzymes and elevated cholestatic liver enzymes, elevated bilirubin and prolonged prothrombin time [5]. Pulmonary complications such as pulmonary hemorrhage with acute respiratory distress syndrome (ARDS) can also occur. In addition, splenomegaly, lymphadenopathy and thrombocytopenia may be found [5]. In this patient, homogenous splenomegaly in addition to perihepatic lymph nodes were noted on CT scan.

Onset of clinical illness occurs abruptly, after an incubation period of 2-20 days (typically 7-12 days) [5]. The acute phase of the illness, called the leptospiraemic phase, lasts 3-9 days and is followed by a period of improvement lasting 1-3 days in which patients become asymptomatic [5]. Thereafter, leptospirosis either regresses to a relatively asymptomatic illness or progresses to a more severe illness [5].

Therefore, leptospirosis should be considered in patients presenting with a fever of unknown origin, particularly when associated with renal failure and jaundice, as a misdiagnosis may be fatal [5]. Thus, once the infection is suspected, the diagnostic evaluation and treatment should be initiated without any delay.

The microscopic agglutination test (MAT) remains the principal test and the gold standard for the diagnosis of leptospirosis [3]. A MAT result is considered positive when titers are > 100 [3]. Serology tests, especially using an enzyme-linked immunosorbent assay (ELISA) method for detecting IgM antibodies, are more frequently used for the diagnosis of Leptospirosis [8]. IgM antibodies usually become detectable five to seven days after the onset of symptoms [2], and a patient is considered positive when a fourfold rise in IgM titers is observed between two samples taken two to four weeks apart [8]. However, these tests are not applicable in the country as they are sent to laboratories located in foreign countries (e.g., France) [3].

Thus, once the infection is suspected, blood samples should be sent for PCR and serology (IgM and IgG) and a follow-up serology should be repeated in 2-4 weeks [8]. In addition, lab tests (CBC, creatinine, thrombin time, cytolytic and cholestatic liver enzymes, bilirubin), chest X-ray and abdominal CT scan should be performed to look for multi-organ involvement.

The organism is often detected in blood through PCR in the first 4 hours to 6 days after symptom onset, while urine PCR typically becomes positive after seven days. PCR has a sensitivity of 40-60% so a negative PCR does not rule out the disease; however, it has good specificity, reaching 95% [10]. In our case, blood PCR did not rule out the disease because it was performed two weeks after the onset of the symptoms, and two days after the beginning of antibiotics.

Patients with mild disease are treated with oral doxycycline (100 mg orally twice daily for seven days), azithromycin (500 mg orally once daily for three days) or amoxicillin (25 to 50 mg/kg in three equally divided doses for seven days, with a maximum of 500 mg/dose). However, patients with severe disease should be started without delay on intravenous third-generation cephalosporin, including ceftriaxone (1 to 2 g IV once daily) or cefotaxime (1 g IV every six hours), or penicillin (1.5 million units every six hours) [11].

However, leptospirosis is often a missed diagnosis because its symptoms often overlap with many other infectious and non-infectious diseases [1].

Clinically, it can be confused with malaria, dengue, chikungunya, scrub typhus, rickettsial infections, typhoid, Hantavirus, and acute viral hepatitis [1]. In addition, leptospirosis may be confused with thrombotic microangiopathies such as thrombotic thrombocytopenic purpura (TTP) and hemolytic uremic syndrome (HUS), which present fever, thrombocytopenia, acute kidney injury, and jaundice. Awareness of these potential mimickers is critical to avoid delays in diagnosis and treatment [12].

## Conclusions

Lebanon’s climate and the presence of carrier rodents provide favorable conditions for the transmission of Leptospirosis. Clinicians should consider the disease in patients with a fever of unknown origin; otherwise, the patient will be mistreated. Empirical treatment should be started once the infection is highly suspected to improve the chances of recovery. Increased awareness and further epidemiological studies are needed to better understand and prevent leptospirosis in Lebanon.
